# HIV service delivery in the time of COVID‐19: focus group discussions with key populations in India

**DOI:** 10.1002/jia2.25800

**Published:** 2021-10-28

**Authors:** Rose Pollard, Usha Gopinath, Yeruva A. Reddy, Bogam R. Kumar, Parthasarathy Mugundu, Canjeevaram K. Vasudevan, Aylur K. Srikrishnan, Aditya Singh, Allison M. McFall, Kenneth H. Mayer, Shruti H. Mehta, Sunil S. Solomon

**Affiliations:** ^1^ Division of Infectious Diseases The Johns Hopkins University School of Medicine Baltimore Maryland USA; ^2^ YR Gaitonde Center for AIDS Research and Education Chennai India; ^3^ Department of Epidemiology The Johns Hopkins Bloomberg School of Public Health Baltimore Maryland USA; ^4^ Department of Medicine Beth Israel Deaconess Medical Center/Harvard Medical School Boston Massachusetts USA; ^5^ Fenway Health The Fenway Institute Boston Massachusetts USA

**Keywords:** COVID‐19, DSD, HIV, India, key populations

## Abstract

**Introduction:**

There are limited data on the impact of COVID‐19‐associated disruptions and novel HIV service delivery strategies among key populations (KPs) in low‐ and middle‐income countries. In March 2020, in response to COVID‐19, the Government of India revised HIV service delivery policies to include community antiretroviral therapy (ART) distribution and multi‐month dispensing (MMD) of ART for all people living with HIV (PLHIV).

**Methods:**

To assess the acceptability of these adaptations and impact of the pandemic among KPs, we conducted focus groups in November–December 2020 with purposively sampled men who have sex with men (MSM), female sex workers (FSWs) and transgender women (TGW) in Telangana and Maharashtra. Seven discussions were conducted. Topics included HIV service access, risk behaviours, economic security and feedback to ensure service continuity. Inductive coding identified themes across topics.

**Results:**

Forty‐four individuals aged 20–49 years participated in discussions (13 MSM; 16 FSW; and 15 TGW). Twenty‐four participants self‐identified as living with HIV. People not living with HIV reported challenges in accessing HIV antibody testing at hospitals due to travel restrictions and fear of contracting COVID‐19. Participants accessed HIV antibody testing using transportation arranged by community‐based organizations after lockdowns eased. PLHIV reported uninterrupted ART refills and generally consistent adherence; however, there were experiences of delayed CD4 and HIV RNA testing. Participants shared appreciation for MMD as it saved time, money, and reduced exposure to COVID‐19. Participants expressed gratitude for home deliveries which enabled ART access, yet shared concerns about home‐based services causing confidentiality breaches with family/neighbours. Participants voiced preferences for community‐based service provision due to proximity, convenient hours, and welcoming environments compared to public hospitals. Other requests included support for income, employment, nutrient‐rich food and more accessible mental health, HIV, and other health services.

**Conclusions:**

COVID‐19 restrictions had a greater impact on access to HIV antibody, CD4, and RNA testing services compared to ART access. High acceptance of MMD and community‐based services support the continued role of differentiated service delivery models to improve KP access to HIV antibody, CD4, RNA testing services, convenient ART retrieval, and integrated services beyond HIV, which may be critical for survival and wellbeing.

## 1 INTRODUCTION

India, with an estimated 2.35 million people living with HIV (PLHIV), bears the third largest burden of HIV globally [1]. HIV prevalence in India is disproportionately higher among key populations (KPs), or groups at higher risk of HIV who often face stigma and criminalization of their behaviours [2]. KPs in India with higher HIV prevalence compared to the general population prevalence of 0.22% include people who inject drugs (6.3%), transgender people (3.1%), men who have sex with men (MSM) (2.7%), and female sex workers (FSWs) (1.6%), based on the last round of national surveillance conducted in 2017 [3]. The national HIV program in India delivers free antiretroviral therapy (ART) from public centres, accessed by KPs and general populations alike. In 2018, India's National AIDS Control Organization (NACO) issued technical ART guidelines, which recommend differentiated care to KPs living with HIV who access ART at public centres [4]. For HIV prevention, India maintains the targeted interventions program through government‐funded, community‐based organizations (CBOs). These programs provide a variety of KP‐focused HIV prevention services, including community‐based HIV and sexually transmitted infection (STI) screening, and commodity distribution (e.g. condoms, lubricant, needles/syringes, and opioid substitution therapy).

The first case of severe acute respiratory syndrome coronavirus 2 (SARS‐CoV‐2) was reported in India on 30 January 2020; the number of cases escalated dramatically thereafter, reaching a peak of almost 100,000 cases per day in September, 2020 before declining. A second SARS‐CoV‐2 wave spiked in March–April 2021, exceeding 400,000 cases per day at its peak [5]. As of 30 June 2021, there were 30,411,634 total reported cases of COVID‐19 in India, the second highest case count globally [5]. The sharp increase in cases during India's first wave was accompanied by a nationwide lockdown that was strictly enforced from March to May 2020. No public transport was operational, and travel was only allowed for essential services during restricted hours. Many government facilities providing HIV services were re‐purposed to provide COVID‐19 case management. Some states continued lockdowns with varying restrictions beyond May 2020. In India's second wave, no national lockdown was instituted but restrictions were regulated on a state‐by‐state basis.

NACO rapidly re‐designed components of their program to ensure service continuity in response to the first wave of COVID‐19. Pre‐pandemic, ART in India was generally dispensed for 30 days through government facilities for all PLHIV, and only PLHIV who met criteria to be considered stable on treatment were eligible to receive multi‐month dispensing (MMD). Prior to September 2018, MMD was approved for 2 months, then switched to 3‐month MMD to be rolled out in phases for eligible PLHIV. As of March 2019, it was estimated that 46% of documented PLHIV on ART in India were receiving MMD [6]. In response to COVID‐19 lockdown restrictions in March 2020, 3‐month MMD became available for all PLHIV. Other policy adaptions in response to the pandemic included expanding home or community‐based delivery of ART rather than facility pick‐up, allowing ART pick‐up from any public ART centre in the country rather than the centre where clients are registered, and issuing multi‐day doses (5–7 days) of opioid substitution therapy [7]. The impact of COVID‐19 and the government's response among KPs is largely unknown, but critical to ensure that gains with respect to HIV/AIDS epidemic control in India are not lost as a result of COVID‐19.

We describe the findings from KP focus group discussions in two high HIV‐burden Indian states to assess the impact of COVID‐19 on access to HIV services among KPs to inform HIV programming and policy.

## 2 METHODS

### 2.1 Study setting

We facilitated KP focus group discussions in November–December 2020 to inform service delivery of a President's Emergency Plan for AIDS Relief (PEPFAR) program working to improve the HIV care continuum among KPs in select districts in the states of Maharashtra and Telangana. These states were chosen because they were identified by PEPFAR as states with high HIV burdens in India; Maharashtra has the highest estimated number of PLHIV in India (396,000) and Telangana has the fifth highest (158,000), as of 2019 [1]. These states together account for about a quarter of the HIV burden in India and contributed 16% of India's newly documented HIV infections in 2019 [3]. HIV transmission in these states is largely sexually driven. Among new HIV diagnoses with self‐reported transmission routes in 2019–2020, 95% were sexually driven in Maharashtra and 97% were sexually driven in Telangana [3]. This is similar to most regions of India apart from the Northeast, where injection drug use is a major driver [3]. As of 30 June 2021, 20% of India's total COVID‐19 cases (30,411,634) were in Maharashtra (6,061,404), the state which bore the highest COVID‐19 burden nationally, and 2% were in Telangana (623,510) [5, 8].

### 2.2 Study population

KPs represented in this sample include MSM, FSW, and transgender women (TGW). Local CBOs who provide services tailored to one of these KP groups facilitated recruitment. They were chosen through a mapping exercise of KP‐focused CBOs in the states of this study. Using a purposive sampling approach aimed at recruiting an information‐rich, balanced sample across KP groups and HIV status [9], program staff worked with the CBOs to identify community members with whom they had existing relationships through service provision. Participants had to be 18 years or older, living in India since lockdowns, and self‐identifying as one of the KP groups of interest.

### 2.3 Study procedures

Semi‐structured interview guides included questions related to four domains: HIV service access, risk behaviours, economic security, and feedback to ensure service continuity. Interview guides were pilot tested and modified accordingly prior to discussions with participants. Due to in‐person COVID‐19 restrictions and to maximize safety, program staff contacted potentially eligible participants by phone to conduct eligibility screening and obtain informed oral consent in the local language of participants. Discussions were organized to have individuals of the same KP group and HIV status as part of the same group. Due to COVID‐19, discussions were either held over the phone using a conference‐calling platform called Voice Snap or in‐person with COVID‐19 safety precautions. For remote discussions, participants called in by phone at the designated time. Facilitators were staff of the program who had experience working with KPs but were uninvolved with CBO service provision. Facilitators were trained in qualitative interviewing, including techniques to encourage full‐group engagement and understanding over the phone, and led each discussion in local languages (Hindi in Maharashtra and Telugu in Telangana). Discussions were also attended by a note taker. Instead of their real names, participants used a pre‐determined unique identification number or pseudonym to identify themselves. A total of seven focus group discussions were conducted with 5–8 participants in each. All participants were compensated 500 Indian Rupees (∼7 USD) for their time.

### 2.4 Data analysis

Audio recordings of discussions were transcribed and translated to English by trained transcribers. One analyst reviewed transcripts on a rolling basis and developed emergent themes, which were reviewed by interviewers and note takers to confirm they represented their understanding of what participants shared. Two analysts developed a codebook using a constant comparison approach [10, 11]. Codes were created inductively from initial transcripts across two a priori categories (experiences and perspectives). Codes were developed by comparing themes within each transcript and subsequent transcripts to determine whether a theme presented a new category, fit an existing category, or added nuance to an existing category. The codebook was added to and refined through this process, aided by discussion between analysts. Next, one analyst applied codes to all transcripts. Then, both analysts independently synthesized themes across codes, comparing similarities and differences between KP groups, HIV status groups, and geographies, and engaged in discussion to clarify findings. Coding was conducted using Dedoose Version 8.0.35 [12].

### 2.5 Ethical clearances

This study was approved by the Johns Hopkins Bloomberg School of Public Health Institutional Review Board (protocol no. IRB00013169), as well the YR Gaitonde Centre for AIDS Research and Education Institutional Review Board (protocol no. YRG 339), the local IRB in India.

## 3 RESULTS

Seven discussions were conducted with 44 participants (Table 1) – four in Telangana (two remotely, two in‐person with COVID‐19 safety precautions) and three in Maharashtra (all remote). Group size ranged from 5 to 8 participants. Thirteen participants were MSM, 16 were FSW, and 15 were TGW; 24 self‐identified as living with HIV. Some MSM and TGW participants also engaged in sex work which became evident through discussion; however, this number was not explicitly documented. The median age of participants was 31 (range: 20–49).

**Table 1:** Focus group discussion participant characteristics

	Total (*n* = 44)	MSM (*n* = 13)	FSW (*n* = 16)	TGW (*n* = 15)
*Age [n(%)]*				
20–29	17 (39)	6 (46)	4 (25)	7 (47)
30–39	18 (41)	3 (23)	9 (56)	6 (40)
40–49	9 (20)	4 (31)	3 (19)	2 (13)
*HIV status [n(%)]*				
Positive	24 (55)	7 (54)	10 (62.5)	7 (47)
Negative	20 (45)	6 (46)	6 (37.5)	8 (53)
*State [n(%)]*				
Maharashtra	18 (41)	6 (46)	6 (37.5)	6 (40)
Telangana	26 (59)	7 (54)	10 (62.5)	9 (60)

Abbreviations: FSWs, female sex workers; MSM, men who have sex with men; TGW, transgender women.

We present themes across the following topics: pandemic impact on sexual behaviours, access to facility‐based HIV testing and treatment services, experiences taking ART, preferences for service delivery, experiences with restricted mobility and limited livelihood, and perspectives about community needs.

### 3.1 Pandemic impact on sexual behaviours

Participants expressed difficulty in finding and meeting sexual partners during the pandemic. Some reported completely stopping sexual activity and others engaged in sex with known partners as they were unable to meet new partners. “During the pandemic, we don't indulge in sex activities that much. We were scared of getting any infection”. (MSM, age 49) Of those who engaged in sex work, including MSM and TGW, there was a decrease in sexual activity during the pandemic, but those who continued sex work or resumed after lockdown reported earning less due to reduced demand, inability to meet clients, and fear of COVID‐19 exposure. TGW in Maharashtra who engaged in sex work reported changes in client interactions since the pandemic, such as clients asking if they have had a COVID‐19 test and requiring them to wear masks during sex. One participant explained how clients now “prefer to have only anal sex because they are scared of getting Corona infection”. (TGW, age 25)

Participants reported no change in condom use during COVID‐19 compared to before. Across groups, participants consistently said that condoms are non‐negotiable for safety. “We make sure that if there is no condom we don't engage in sexual activities. I feel that condom is most important”. (MSM, age 35) MSM in both states reported a lack of reliable condom stock at public hospitals during COVID‐19. However, all KP groups reported that CBOs helped maintain their supply of condoms.

### 3.2 Access to facility‐based HIV testing and treatment services

Disruptions from COVID‐19 heightened several barriers for participants trying to access facility‐based services for HIV antibody, CD4, and HIV RNA testing, compared to before the pandemic. Participants reported barriers to travel to facilities to get an HIV test or pick up ART, and confusion over which clinics were open or still offering these services given that hospitals had transitioned to treating COVID‐19 patients:

*Other health services were put on a back foot in front of COVID‐19. I was willing to get my HIV test done but transport service was shut. So, in spite of having biannual HIV test due, I did not get tested and I felt that I shouldn't have missed it. (MSM, age 35)*

Participants also mentioned avoiding HIV antibody testing because “there are so many Corona cases in government hospitals”. (TGW, age 25) After lockdowns eased, some accessed HIV antibody testing through support of CBOs who helped make appointments and assist with transportation.

Disruptions in mobility and at health facilities resulted in delayed CD4 and HIV RNA tests for PLHIV. Hospitals cancelled CD4 test appointments or deferred them until after lockdown for multiple participants. One MSM, age 29, shared that he completed CD4 and HIV RNA testing at a public hospital post‐lockdown, but experienced a delay in getting his results. Similar to HIV antibody testing support, CBO staff helped by providing transportation or accompanying participants over the pandemic to CD4 test appointments at public hospitals.

### 3.3 Experiences taking ART

There were reported challenges in taking ART regularly during the pandemic among participants, most notably when living with family over lockdown:

*I had faced problems while taking the medicines because my brothers were asking me, what are these medicines for and why I was taking them. I had to lie to my family members about the medicines. I also had difficulty in keeping the medicines at home. (MSM, age 40)*

However, participants living with HIV generally reported taking ART regularly during COVID‐19 without lapses in adherence.

Participants retrieved refills of ART either in‐person at public ART centres or through home deliveries from CBOs. There were a few challenges in ART pick‐up. One participant did not take ART for 15 days during lockdown after she ran out of pills, “I had gone to get my medicines, but they said that there is a shortage of medicines. So, I had to wait until the stock arrived”. (FSW, age 30)

Despite these challenges, participants reported that new support mechanisms helped sustain ART access over the pandemic. KPs in both states reported receiving door‐delivery of ART and expressed gratitude for the service, as it enabled them to maintain their stock. TGW in Maharashtra shared how a CBO in their area contacted them directly to ask about ART adherence and helped get them ART if needed. One participant described how his local CBO proved helpful especially after he tested positive for COVID‐19:

*I am thankful to them. As per medicines, I did not face any problems…[NGO name] delivered 3 boxes [of ART] to my house when I had informed that I have medicine shortage. By then I was COVID positive, they told me that there is no need to go out and delivered my medicines. (MSM, age 24)*

A major change for participants living with HIV over the pandemic was receiving MMD, both through pick‐ups and door deliveries. Participants across KP groups appreciated MMD, mentioning how it reduced trips to hospitals, saved money on travel expenses, and reduced disruptions in daily life, such as missing work:

*It would be helpful if medicines are given for three months at a time. As we do private jobs, every month they might not give permission to go and get our medicines. They might have doubts that why are we asking permission every month on that particular date. (MSM, age 26)*

Participants also shared concerns with MMD, mentioning that it could make it harder to keep their status a secret from others:

*Taking medicines once in a month is good because if we have a stock of three months medicines, it will be difficult to hide them. What if someone sees them and tells others? If it is a single box with one month's medicines, it will be easy to hide. (FSW, age 30)*

Participants reported misconceptions related to HIV, ART, and COVID‐19. These included the idea that taking ART mitigated the risk of contracting SARS‐CoV‐2 infection, and living with HIV increased susceptibility to infection. Participants living with HIV shared how they were fearful of exposure especially as a person who is HIV positive, “We are at least living with HIV, if we get COVID we might perish. I am not sure if we will ever get treatment for it”. (FSW, age 35) This fear made some question the safety of going to clinics to collect their ART.

### 3.4 Preferences for HIV service delivery

Participants said that they would prefer to access services across the HIV cascade (i.e. HIV antibody testing, ART pick‐up, CD4 and HIV RNA testing) through CBOs compared to public hospitals or clinics due to proximity, extended hours, and more welcoming environments. One participant shared his view that CBOs are well‐placed to distribute ART and support on‐time pick‐ups compared to public hospitals:

*[CBOs] have good accessibility and it is easier for them to do tracking. They can call up the members and remind about the medicine due date…It is better to hand over the responsibility to the community than going to [public hospital] and standing in the queue. (MSM, age 24)*

Participants also preferred going to community‐based service locations to avoid stigmatizing environments in public clinics. TGW in Telangana not living with HIV shared that they experience “odd looks” and “teasing” at public hospitals and staff do not take their health concerns seriously, so going to a CBO for HIV antibody testing is better than going to the hospital.

Participants shared conflicting opinions about home‐based services initiated during lockdowns. There was appreciation for the convenience of home‐delivery of ART and the perspective that this service delivery should continue. However, when sharing perspectives about whether or not other services should hypothetically be delivered at home in the future, such as HIV antibody testing or CD4 testing, confidentiality concerns arose:

*If they come home for [CD4] testing, the whole world will know about it…if the house owner comes to know about it, he will throw us out and nobody will give house for rent…Then we will have to shift to the forest. (FSW, age 35)*

*We do not want [CD4] testing conducted at home…Most of us live with our family members and we do not want our family members to know about the testing. (MSM, age 24)*

Participants were open to the idea of telemedicine consultations (by phone or video call) as it saved time and money; however, there were reservations. One MSM, age 35, thought that a patient needs to meet a doctor physically to get better treatment. FSW and TGW both expressed concerns related to technology access, as many in their communities do not own a computer or smartphone.

### 3.5 Experiences with restricted mobility

Difficulty to travel and move around during the pandemic emerged as a prominent theme across topics for KPs. Participants reported staying inside during lockdowns, as public transit was inaccessible and curfews were enforced. Restrictions affected participants” access to healthcare and resulted in a lack of clarity as to which pharmacies or service venues were operational. Participants either experienced or heard about harassment from police for travelling during lockdowns, which led to fear of leaving the house, “Females due to fear were not ready to go [to the hospital]…they used to say that police beat a lot…why unnecessarily get beat by police and come home?” (FSW, age 34)

### 3.6 Experiences with limited livelihood

Another salient theme was how disruptions from the pandemic reduced income for participants, which caused stress and challenges to cover basic needs, including food and rent:

*We used to have good food and be healthy. It used to [be] sufficient for us to survive. At times I also used to go to work. Now there is no money, [I] have taken loans and repaying them is difficult. We are facing lot of problems. (FSW, age 30)*

Prior to the pandemic, participants earned income from a variety of activities, including agriculture, daily wage jobs, and sex work. These income sources were far less lucrative over the pandemic, as earning opportunities reduced, travel was difficult, and activities produced less earnings. TGW reported challenges to earn money from begging, an important source of income in some TGW communities, “I go for begging at the signals, the vehicles are not giving us money…earlier we used to get 1,000 to 1,500 [rupees per day], now we get 200 to 300. It has become very difficult”. (TGW, age 20)

Participants found alternative sources of income, such as this MSM, age 35, “We were not working during the lockdown. I learned how to run the sewing machine so I made masks to sell, and I earned a subtle income”. Across groups, financial insecurity emerged as an ongoing point of stress post‐lockdowns for participants and their communities.

### 3.7 Perspectives about community needs

When discussing needs, participants requested help to find income opportunities, support to access government pensions they may be eligible for, skills training to find employment, and provision of nutrient‐rich food or supplements for themselves and their families. One TGW described the impact which employment support could have in her community:

*There are well‐educated TG people who are getting decent jobs, and there are illiterate people with other skills sets. So if we get the proper opportunity, we can bring changes in our own life and stop begging and sex work. (TGW, age 36)*

Other trends for service priorities included COVID‐19 testing, COVID‐19 vaccine provision and mental health counselling. Participants also requested increased availability of HIV antibody testing, CD4 and HIV RNA testing, and accessible ART. Suggestions to make these services more available included subsidized or free travel to get to clinics for HIV‐related services, and a mobile van to deliver ART and collect blood samples near people's homes.

## 4 DISCUSSION

This qualitative assessment explored the impact of COVID‐19 on HIV‐related behaviours and HIV prevention and treatment access among MSM, TGW, and FSW in the high HIV‐burden Indian states of Maharashtra and Telangana. We found that participants were appreciative of adaptations of the national AIDS program to ensure continuity of services, such as MMD and home/community‐based ART delivery; however, participants encountered barriers to access facility‐based testing services (HIV antibody testing as well as CD4 and HIV RNA testing) throughout the pandemic. A recurrent theme was the impact of COVID‐19 on livelihood, which led to concerns with respect to securing food and housing.

Participants reported fewer sexual partners during the pandemic and tended to use condoms during sex, which may imply decreased HIV risk. In an online study in the United States, most MSM reported having the same or fewer sexual partners early in the pandemic, but 1% did increase their number of partners, and about a quarter indicated increased alcohol or other recreational drug use [13]. A different survey with MSM in the United States contrastingly found that MSM on average increased their number of sexual partners over the COVID‐19 lockdown period, and those with increased substance use were significantly more likely to report increases in number of sexual partners [14]. Both surveys found that MSM maintained their pre‐COVID condom usage, in parallel with participants in our study. More research is needed to ascertain the impact of COVID‐19 on HIV risk among KPs by investigating changes in sexual behaviours and substance use over the course of the pandemic.

Accessing facility‐based testing services (i.e. HIV antibody and RNA testing) was a challenge for participants in this study. This experience is not unique to KPs in India. Emerging data on the impact of COVID‐19 among MSM in various sites illustrate how HIV antibody testing has been harder to access [13, 15, 16], which was also seen in the United States, as the pandemic caused interruptions and declines in HIV/STI testing access [17]. HIV programs globally saw fewer clients living with HIV complete HIV RNA testing over the initial months of COVID‐19 compared to pre‐pandemic [18]. Public health programs can help restore testing service utilization by exploring innovative solutions, such as delivering testing through community health workers or incorporating HIV self‐testing into service delivery [19, 20, 21]. These strategies may be particularly important to maintain access to HIV diagnosis and RNA testing for KPs, who already face socio‐structural access barriers, especially if travel or facility disruptions from COVID‐19 continue.

Key barriers to ART pick‐up reported by our participants are consistent with those reported from adults on ART in Kampala, Uganda, who reported that stay‐at‐home orders negatively impacted ART access due to transportation challenges, police violence, and fear of COVID‐19 [22]. Despite these barriers, participants living with HIV in our discussions were overall able to maintain ART adherence. This speaks to the pandemic response of NACO to limit trips to routine ART distribution sites through MMD and expanded community‐based outreach, and highlights the critical role of CBOs. Differential access to HIV testing and treatment services has also been observed in countries with generalized epidemics. A study in South Africa examining HIV service access across 65 clinics during the pandemic found that HIV antibody testing was more heavily impacted than ART provision [23]. Another study assessing the effect of COVID‐19 on 1,059 health facilities in 11 African countries observed that HIV antibody testing decreased, but MMD and ART home delivery likely enabled ART adherence [24].

Adapting how HIV services are delivered to the unique needs of each person, or differentiated care, can help ensure uninterrupted service access for KPs as COVID‐19 disruptions continue. Strong preferences among our participants, especially around door‐deliveries and community‐based HIV service delivery, highlight the importance of tailoring services to individuals' preferences and context [22, 25]. These findings also re‐affirm that “one size does not fit all”, as evident from varied reactions to MMD and telemedicine. While the rapid transition towards virtual service delivery, MMD, and field‐delivery of ART in response to the pandemic is a major step towards client‐centred, decentralized HIV care, it is crucial to implement these approaches with the ability to tailor options to individual preferences [26, 27].

Our findings can inform guidelines and policies which help expand community‐based service provision and facilitate service access for KPs. As community‐based ART dispensation models have been expanded over COVID‐19 in India, developing guidelines for community service provision can facilitate standardized implementation and scale‐up of such models at the district‐level. These policies should incorporate recommendations to tailor delivery models to various KP groups and contexts by gathering community input, and accommodating preferences and concerns surrounding confidentiality.

Most HIV programs in India and globally have a vertical programming structure with the objective of delivering optimal HIV‐associated services. The COVID‐19 pandemic highlights the need to design client‐centred programs and re‐think vertical programming to integrate comorbidities, such as mental health and non‐communicable diseases [28]. Our findings that COVID‐19 exacerbated challenges for KPs to access basic resources are consistent with other settings [13, 29, 30]. Since basic needs, including housing and food, come before access to healthcare for many, HIV programs and policies should think more comprehensively for the benefit of KPs to include access to social support services in such times of public health emergencies. This is in line with recent calls for HIV programs to move towards comprehensive care, rather than a single‐disease approach [31, 32]. Catering to social determinants of health and people's most pressing needs may engage more people in services and contribute to favourable outcomes across the HIV cascade for KPs [33]. Policies and program approaches would benefit from further research investigating variations of service access and preferences across KP groups in India, especially those related to community‐based service modalities and comprehensive care.

There are limitations to this study. Participants were recruited through CBOs which may limit generalizability to KPs who are not engaged in services. As opposed to in‐person focus groups with face‐to‐face interaction, focus groups in our study held over the phone presented some challenges to natural conversation and rapport‐building. In these remote discussions, participants could not see each other and there were a few instances where participants experienced phone connectivity issues. However, at the time of data collection, remote interaction was necessary as per local government restrictions and to prevent the spread of SARS‐CoV‐2. Although our sample size is small if disaggregated per KP group, our purposive sampling approach worked to optimize for information power and variation of KP group while considering implementation feasibility [34]. Our study did not recruit people who inject drugs, a group at higher risk for HIV infection in India. As the majority of HIV infections in India are sexually driven, especially in the states of Maharashtra and Telangana, it was challenging to incorporate people who inject drugs in our focus groups. Therefore, it is possible that people who inject drugs were impacted by COVID‐19 in ways that are not captured in this manuscript. Also, as sex work was not the primary focus of our study, more research is needed to make direct inferences about the impact of COVID‐19 on experiences with sex work among KPs in India. Although our study was only conducted in two Indian states, Maharashtra and Telangana are well‐placed to represent other high‐HIV burden states across India, except for the Northeast where HIV transmission is disproportionately driven by injection drug use, since public HIV services across India follow standardized national guidelines. While findings are likely not representative of KPs across all of India or globally, our study offers insight into the experiences and perspectives of KPs given the pandemic in order to strengthen HIV prevention and treatment services.

## 5 CONCLUSIONS

As COVID‐19 continues to impact health services, ensuring continuity in HIV preventive and treatment services is paramount to maintain and build on progress made in the past two decades towards HIV epidemic control. This is especially needed for KPs in low‐ and middle‐income countries, for whom disruptions from COVID‐19 threaten to widen existing economic and societal disparities compared to general populations. Our findings support the need for differentiated service delivery to bridge gaps of access to facility‐based testing, integrate comprehensive care with HIV services, and expand community‐based services in ways that remain sensitive to individual preferences and varying community and environmental contexts.

## COMPETING INTERESTS

SSS received consulting fees and research grants and products for his institution from Gilead Sciences and research grant and product from Abbott Laboratories outside of the submitted work. SSS and SHM received consulting fees from Gilead Sciences outside of the submitted work. KHM has received research grants outside of the submitted work for his institution from Gilead, Merck and Janssen, and has served on scientific advisory boards for Gilead, Merck and ViiV focused on HIV prevention.

### AUTHORS' CONTRIBUTIONS

AMM, SHM, SSS and RP designed the study, developed the data collection tools, and trained field teams. YAR, BRK, PM, CKV, AKS and AS led and supervised data collection. UG and RP conducted data analysis, and RP drafted the manuscript with key inputs from KHM and SSS. All authors read and approved the final manuscript.

## FUNDING

This project has been supported by the U.S. President's Emergency Plan for AIDS Relief (PEPFAR) through a cooperative agreement through the United States Agency for International Development (USAID) under the terms of cooperative agreement #72038619CA00001.
